# Development and Characterization of a Human Mammary Epithelial Cell Culture Model for the Blood–Milk Barrier—A Contribution from the ConcePTION Project

**DOI:** 10.3390/ijms252111454

**Published:** 2024-10-25

**Authors:** Debora La Mantia, Nina Nauwelaerts, Chiara Bernardini, Augusta Zannoni, Roberta Salaroli, Qi Lin, Isabelle Huys, Pieter Annaert, Monica Forni

**Affiliations:** 1 Department of Veterinary Medical Sciences, University of Bologna, Ozzano dell’Emilia, 40064 Bologna, Italy; chiara.bernardini5@unibo.it (C.B.); augusta.zannoni@unibo.it (A.Z.); roberta.salaroli@unibo.it (R.S.); 2 Drug Delivery and Disposition Lab, Department of Pharmaceutical and Pharmacological Sciences, KU Leuven, O&N II Herestraat 49—Bus 921, 3000 Leuven, Belgium; pieter.annaert@kuleuven.be; 3 Health Sciences and Technologies-Interdepartmental Center for Industrial Research (CIRI-SDV), Alma Mater Studiorum—University of Bologna, 40126 Bologna, Italy; monica.forni@unibo.it; 4 BioNotus GCV, Galileilaan 15, 2845 Niel, Belgium; qi.lin@bionotus.com; 5 Department of Clinical Pharmacology and Pharmacotherapy, KU Leuven, ON II Herestraat 49—Bus 521, 3000 Leuven, Belgium; isabelle.huys@kuleuven.be; 6 Department of Medical and Surgical Sciences, University of Bologna, 40138 Bologna, Italy

**Keywords:** human mammary epithelium, primary cell culture, blood–milk barrier, breastfeeding, transepithelial electrical resistance, in vitro barrier model, membrane transport proteins, cell membrane permeability

## Abstract

It is currently impossible to perform an evidence-based risk assessment for medication use during breastfeeding. The ConcePTION project aims to provide information about the use of medicines during lactation. The study aimed to develop and characterize an in vitro model of the blood–milk barrier to determine the extent of the milk transfer of xenobiotics, relying on either on human mammary epithelial cells (hMECs) or immortalized cell lines derived from breast tissue. The hMECs were cultured and characterized for epithelial markers; further, the ability to form an epithelial barrier was investigated. Drug transporter functionality in the cultured hMECs was analyzed with specific probe substrates. The hMECs showed an epithelial morphology and the expression of epithelial markers and tight junctions. They formed a reproducible tight barrier with a transepithelial electrical resistance greater than 400 Ωcm^2^, unlike immortalized cell lines. Different levels of mRNA expression were detected for 81 genes of membrane transporters. Functional assays showed no evidence for the transporter-mediated secretion of medicines across the hMECs. Nevertheless, the hMEC-based in vitro model covered a 50-fold range of permeability values, differentiating between passive transcellular and paracellular-mediated transport. The cultured hMECs proved to be a promising in vitro model for biorelevance; the wide characterization of hMECs makes them useful for studying medicine partitioning in milk.

## 1. Introduction

Breastfeeding is associated with health benefits for mothers and infants. The World Health Organization (WHO) recommends on demand exclusive breastfeeding for 6 months, and continued breastfeeding for at least two years. At the same time, more than 50% of women require medicine in the postpartum period [1]. Unfortunately, there is uncertainty about the exposure-related safety for the breastfed infant of most medicines used by lactating mothers [2]. Clinical studies in this vulnerable population pose ethical and practical challenges. Therefore, non-clinical methods carry the promise to accelerate data generation in this domain. Several models have been developed to predict the milk-to-plasma ratio (M/P ratio) based on physicochemical properties [3,4,5], but these models often face over- or underprediction for specific physicochemical classes of medicines. Moreover, physicochemical-based models are currently not capable of predicting transporter-mediated processes between maternal blood and milk. In vitro models have been explored to predict the transfer of medicines across the human blood–milk barrier, based on both primary cells and immortalized cell lines in animal and human cell culture models [6]. Rodent in vitro models have been used to estimate drug transport across the mammary epithelium [7,8], but they are not representative of the human mammary epithelium due to species differences [9]. Yang et al. (2022) developed an in vitro in vivo extrapolation model (IVIVE) to predict the milk-to-plasma (M/P) ratio based on the efflux ratio determined in an immortalized cell line of human colorectal adenocarcinoma Caco-2 [10]. The Caco-2 model offers the advantage that monolayer permeabilities are available for a wide range of compounds. However, this established in vitro model is inaccurate for certain transporter classes (e.g., P-glycoprotein (P-gp)) and is not representative of in vivo mammary physiology. Different mammary tissue-derived cell lines have been used to examine the transport of small organic molecules. Andersson et al. (2017) studied the transfer of the neurotoxic amino acid β-N-methylamino-alanine (BMAA) in the breast cancer epithelial MCF-7 cell line [11]. Other human cell lines have been investigated for mechanisms involving the normal physiology of the mammary gland. PMC42-LA [12] is a mesenchymal breast carcinoma cell line that has been described to represent a lactating state in the presence of prolactin, whereas MCF-10A [13] is an immortalized non-tumorigenic cell line typically used as in vitro model for the normal breast, but none of these studies provided information on the ability to study the secretion of medicines into human milk. The MCF-10F cell line-based in vitro model developed by Zhang et al. (2022) showed promising results for the prediction of the M/P ratio [14]. They showed that MCF-10F permeability data improved M/P prediction compared to the Caco-2 cell line for four P-gp substrates. The use of primary human mammary epithelial cells (hMECs) to study the transfer of medicines into human milk has first been described by Kimura et al. (2006), where a tight monolayer was obtained after three trypsin treatments where the cells that remained attached (about 20%) were allowed to continue growing on the same dish. Primary cell cultures better preserve the in vivo condition compared to immortalized cell lines, whose use in biomedical research has intrinsic limitations due to genotypic and phenotypic drifts [15]. For this reason, primary cells could offer a more predictive in vitro model for determining human M/P ratios. Primary cultures also offer the possibility of representing natural population variability through true biological replicates, or the use of pooled cultures of multiple subjects.

The present study fits within the Innovative Medicine Initiative (IMI) project ConcePTION, launched in April 2019, which aims to reduce uncertainty about medicine use during pregnancy and lactation encouraging the development of an in vivo, in vitro, and in silico model. Previously, we developed a pig and minipig in vitro model of the mammary epithelial barrier [16,17], based on the assumption that the pig/minipig was selected by the ConcePTION consortium as an animal model for in vivo lactation studies [9]. Concurrently, a well-characterized human mammary epithelial cell model is necessary to predict the drug distribution in milk, to further evaluate the in vitro correlation between the porcine and human models in a translational perspective. The aim of the present study was to develop and characterize a human cell-based in vitro model capable of quantifying the transfer of xenobiotics across the blood–milk barrier. In the present study, both primary mammary epithelial cells (hMECs) and immortalized cell lines (MCF-7, MCF-10A and PMC42-LA) were evaluated for this purpose.

## 2. Results

### 2.1. Selection of Human Cell Culture Models

The hypothesis was that the epithelial cells form the main barrier for medicines between the blood and human milk. Therefore, both cell lines (MCF-7, MCF-10A and PMC42-LA) and primary hMECs were considered as the in vitro model for the blood–milk barrier. Representative images of the cell lines are shown in Supplementary Figure S1.

### 2.2. Barrier Function of Cell Lines

**Figure 1:**
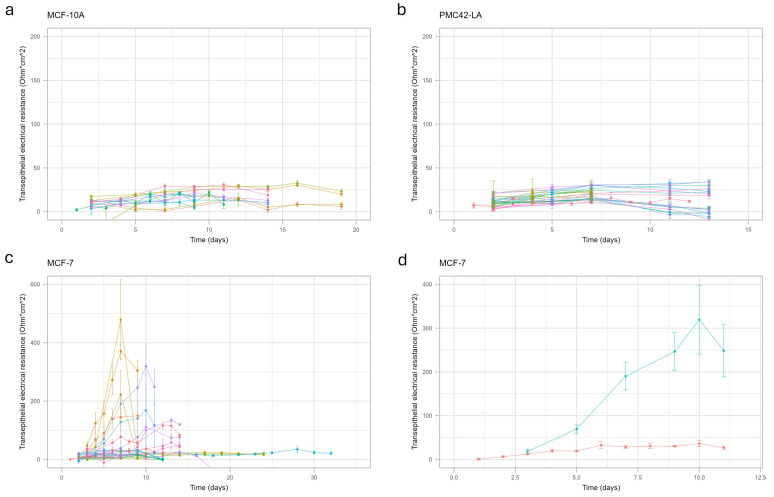
Transepithelial electrical resistance (TEER) of immortalized cell lines under explored culture conditions measured over time: MCF-10A (**a**), PMC42-LA (**b**), MCF-7 (**c**,**d**) MCF-7 cells (independent replication represented in red and blue) were cultured in full growth medium without beta-estradiol containing 5% fetal bovine serum seeded at 2 *×* 10^5^ cells/cm^2^ on polyethylene terephthalate (PET) transwell inserts of 0.4 µm membrane pore size (**d**). Points represent mean values; error bars represent standard deviation; different colors represent explored culture conditions (details are shown in Table S1).

The barrier function of human cell lines (MCF-10A, PMC42-LA and MCF-7) was evaluated via the measurement of transepithelial electrical resistance (TEER) and transport of sodium fluorescein across the cells grown on transwell inserts. The explored conditions did not result in a tight barrier, as shown by transepithelial electrical resistance for MCF-10A (Figure 1a) and PMC42-LA (Figure 1b) cell lines, and also confirmed via sodium fluorescein leakage (Supplementary Table S1). Although occasionally a tight barrier was obtained for MCF-7 cells (Figure 1c), these results could not consistently be replicated with identical culture conditions (Figure 1d). Since the cell lines did not form a barrier suitable for permeability experiments under the explored conditions, further characterization was only performed for hMECs.

### 2.3. Morphology of Primary hMECs

**Figure 2:**
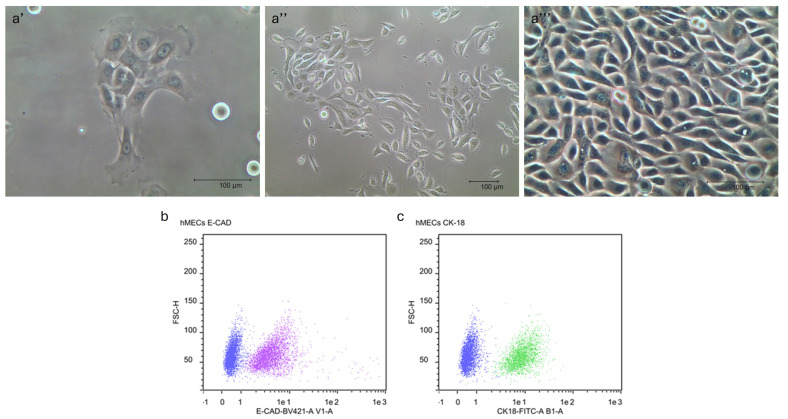
Representative images of human mammary epithelial cells (hMECs) at 30% confluence from seeding showing the typical epithelial morphology and growth in cluster, 20× objective (**a’**), 10× objective (**a’’**), cells at around 80% confluence reached in one week, 20× objective (**a’’’**). Scale bar 100 μm. Representative images of flow cytometric analysis of E-cadherin (E-CAD) (**b**) and cytokeratin-18 (CK-18) (**b**,**c**) in hMECs when reaching 80% confluence. Overlay between unmarked sample (in blue) and samples marked with E-CAD (in violet) and with CK-18 (in green).

Primary cell cultures of hMECs showed a growth in clusters and exhibited a cobblestone-like epithelial morphology, reaching confluence within a week (Figure 2a’–a’’’). The mean doubling time was 36.6 ± 3.25 h.

### 2.4. Immunophenotypic Characterization by Flow Cytometry Analysis (FC)

The quantitative flow cytometry single-cell analysis revealed that hMECs cultures expressed the cell adhesion protein E-cadherin (E-CAD, Figure 2b) and the mammary epithelial-specific intermediate filament protein cytokeratin-18 (CK18, Figure 2c). Both CK18 and E-CAD positive peaks exhibited bright fluorescence intensity, indicating positivity throughout the entire cellular populations.

### 2.5. Barrier Function of Primary hMECs

**Figure 3:**
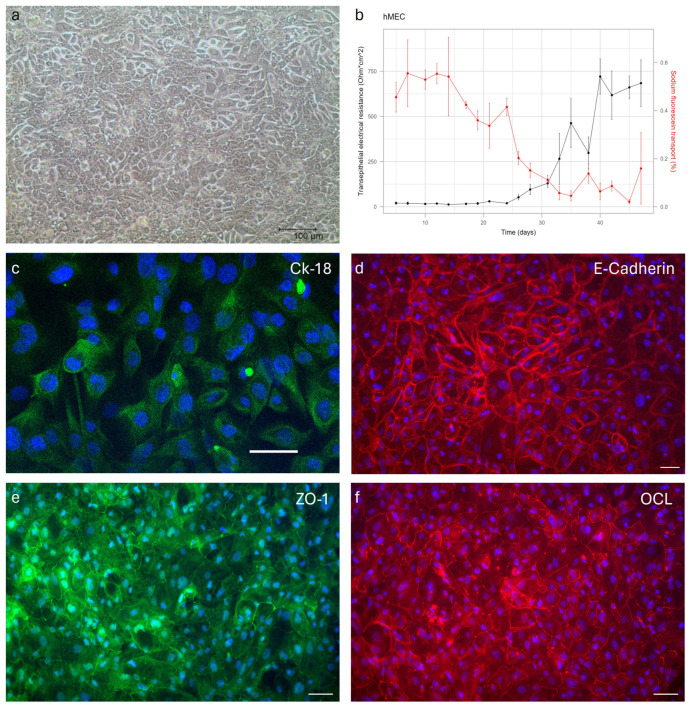
Monolayer integrity of primary human mammary epithelial cells (hMECs). hMECs grow compact on transwell inserts (**a**). Transepithelial electrical resistance (TEER, Ohm × cm^2^) graph in black and sodium fluorescein transport (%) in red (**b**). Points represent mean values; error bars represent standard deviations. Immunofluorescence staining of hMECs showing the expression of epithelial markers: cytokeratin-18 (CK18) and E-cadherin (E-CAD) (**c**,**d**), and the expression of tight junctions: zonula occludens-1 (ZO-1) and occludin (OCL) (**e**,**f**) after 35 days of culture on transwell inserts. Scale bar 50 μm.

The barrier function of hMECs was explored under different culture conditions (Supplementary Table S1). The hMECs formed a tight barrier when 10% fetal bovine serum (FBS) was added to the medium after ±35 days of culture on polyethylene terephthalate (PET) transwell inserts with a pore size of 0.4 µm and a surface area of 0.3 cm^2^ growing as a compact monolayer (Figure 3a). The maximum TEER value was 721 ± 97 Ω × cm^2^ after 40 days. The sodium fluorescein leakage reached a minimal value of 0.021 ± 0.009% after 45 days (Figure 3b). The formation of a tight barrier at 35 days was also confirmed using an immunohistochemical staining of tight junction proteins.

The immunofluorescent (IF) analysis of cellular monolayers of hMECs, cultured on transwell inserts for 35 days, evidenced the expression of epithelial markers, the mammary epithelial-specific intermediate filament protein CK18, and the cell-adhesive protein E-CAD (Figure 3c,d). Moreover, the hMECs created a compact monolayer on the transwell inserts, resulting in the expression of tight junctions zonula occludens-1 (ZO-1) and occludin (OCL) (Figure 3e,f).

### 2.6. Membrane Transporters in hMECs

**Figure 4:**
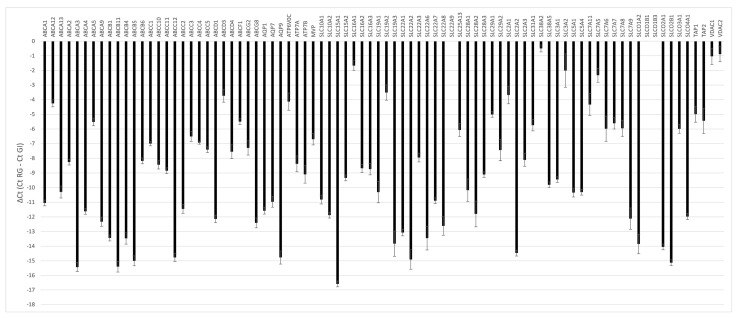
mRNA array analysis of transporters present in human mammary epithelial cells (hMECs). The genes analyzed are represented as relative expression calculated as ∆Ct (mean Ct reference genes (RG)—Ct gene of interest (GI)) ± SD (*n* = 3).

The membrane transporters in the hMECs were evaluated based on an mRNA array study and the permeability of probe substrates. The transcriptional profile of drug transporters showed a differential level of gene expression (Figure 4), ranging from ΔCt values that were very negative (lower expression) to ΔCt values that were less negative (higher expression). Among the eighty-four analyzed genes, only three were not detectable (*SLC22A9*, *SCLO1B1*, and *SLCO1B3*).

**Figure 5:**
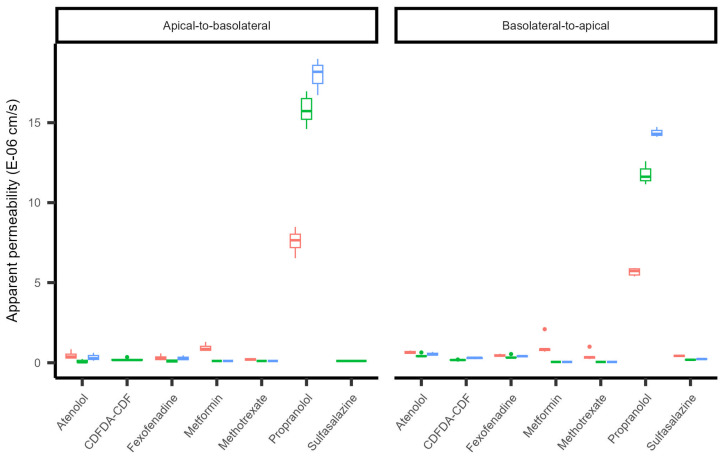
Permeability of probe substrates for transport routes in human mammary epithelial cells (hMECs). Apparent permeability (Papp, 10^−^^6^ cm/s) of probe substrates for functionality of transport routes in hMECs. Three independent experiments with each three or six technical replicates are shown in red (*n* = 6), green (*n* = 6), and blue (*n* = 3). Boxplots represent median and interquartile range; lines represent the range and points represent potential outliers. 5(6)-Carboxy-2,7-dichlorofluorescein diacetate (CDFDA) is hydrolyzed to 5-(and-6)-carboxy-2′,7′-dichlorofluorescein (CDF) via intracellular esterases. The apparent permeability was calculated using the cumulative amount of CDF transported in function of time and the donor concentration of CDFDA. The apical-to-basolateral apparent permeability for CDFDA-CDF (green) was estimated from only two time points (i.e., linearity could not be evaluated), as the earlier time points were below the limit of quantification.

The hMEC in vitro model showed higher bidirectional apparent permeability for propranolol compared to atenolol, confirming the ability to distinguish between passive paracellular and transcellular transport routes (Figure 5, Table 1). The bidirectional apparent permeabilities of the probe substrates for membrane transporters were very low, and showed limited polarity, suggesting limited membrane transporter functionality in the hMEC in vitro model under the conditions tested (Figure 5, Table 1).

## 3. Discussion

Considering the scarce scientific evidence on safely using medicines by pregnant and lactating women, the funded European project ConcePTION [18] (https://www.imi-conception.eu/) supported and encouraged the development and validation of non-clinical in vitro, in vivo, and in silico models to predict the passage of medicines across the blood–milk barrier [6,19]. In the present study, we developed an in vitro model to determine the transfer of medicines across the blood–milk barrier, based on the assumption that mammary epithelial cells form the main barrier between plasma and human milk. To better preserve in vivo epithelial phenotypes and improve studies involving the in vitro to in vivo correlation (IVIVC), primary hMECs were used as the most biorelevant model. In addition, given the limited lifespan of primary cells, human immortalized cell lines, like MCF-7, MCF-10A, and PMC42-LA, were also evaluated. The in vitro model allows us to measure bidirectional permeability coefficients across the human epithelial barrier. Culture conditions were optimized to obtain a tight monolayer of cells cultured on semipermeable transwell inserts. Although more advanced in vitro models could be explored, the current approach carries the promise to combine experimental simplicity (feasibility) with predictive potential. A similar approach is widely applied to calculate the permeability of medicines in the blood–brain barrier or at the intestinal epithelium using Caco-2 cells [20,21].

In the current study, cell lines (MCF-7, MCF-10A, and PMC42-LA) did not form a tight monolayer suitable for permeability experiments under the explored culture conditions. Marshall et al. (2009) have previously described the formation of a tight monolayer of the MCF-10A cell line by the removal of cholera toxins from the medium [22]. Other reports described the absence of tight junctions in this cell line [23,24], similar to the results obtained here. No information was found in the literature about the ability of the PMC42-LA cell line to form a tight barrier. For MCF-7, some reports mention the formation of a tight barrier [25,26], whereas others report low transepithelial electrical resistance values [27,28]. We found initially that MCF-7 cells were able to form a tight barrier when beta-estradiol was omitted from the medium during several passages in culture, but these results could not be consistently reproduced.

In the present research, the culture of primary hMECs showed that cells maintained an epithelial morphology until passage 10, which was also confirmed by the flow cytometry analysis on the expression of epithelial markers E-CAD and CK18. Kimura et al. (2006) first described the formation of a tight barrier by a trypsin-resistant population of hMECs in the presence of prolactin [29], but we were unable to replicate these results. Zhang et al. (2022) also reported that they failed to replicate the results from Kimura et al. (2006) [14]. This inconsistency could be due to the high variability inherent in the primary cell culture [30]. In fact, in our model, the number of trypsin treatments or addition of prolactin did not seem to positively impact the barrier formation. However, the addition of 10% FBS to the culture medium resulted in the formation of a tight barrier after ±35 days. This is a relatively long culture time, especially compared to the (mini)-pig mammary epithelial cell in vitro models that have been described previously [16,17]. Nevertheless, the epithelial cell type and formation of tight junctions after this culture time was confirmed via immunohistochemical staining of epithelial markers and tight junction proteins.

The primary mammary epithelial cells are the preferred cells in light of the objective of this study, as they result in the most biorelevant model, but also have the ability to form a tight barrier suitable for permeability experiments. Indeed, the bidirectional permeabilities for propranolol were high in comparison to atenolol, confirming that the in vitro model is able to distinguish between passive paracellular and transcellular transport routes.

To better characterize the hMEC in vitro model, real-time PCR testing was used to screen for the expression of a panel of 84 different transporter gene transcripts. Of the eighty-four genes analyzed, eighty-one were detectable with a different mRNA expression level, while only three genes were not detectable: *SLC22A9*, *SCLO1B1*, and *SLCO1B3*. For comparison, a previous study that investigated transporter gene expression (30 genes) in human epithelial cells was performed under different conditions. In that study, non-lactating and lactating epithelial cells were purified, respectively, from breast tissue or milk, without establishing an in vitro culture system [31].

The organic cation transporter (OCT) and organic anion transporter (OAT) gene subfamilies belong to the solute carrier transporter (SLC) superfamily. Among the SLC22 transporters, we showed high expression only for OCT3 (*SLC22A3*). Furthermore, we found very low levels of expression for the other OCT genes. Partially in agreement with our results, Alcorn et al. (2002) found OCT1/3 gene expression, with increased RNA levels of OCT1 (*SLC22A1*) in lactating compared to non-lactating mammary epithelial cells, and no gene expression of OCT2 [31]. Within the OAT subfamily, we showed expression, even if very low, of OAT1 (*SLC22A6*), OAT2 (*SLC22A7*), and OAT3 (*SCL22A8)*, while these genes were below the detection level in non-lactating cells according to Alcorn et al. (2022) [31]. OAT4 (*SLC22A9*) was not detectable. In agreement with the gene expression data, we found very low bidirectional apparent permeabilities for metformin (OCT substrate) and methotrexate (OAT substrate), with most of the samples in the receiver compartment below the lower quantification limit (5 nM). In agreement with the literature, among other SLC transporters analyzed (*SLC15A1*/PEPT1; *SLC15A2*/PEPT2; *SCL28A1*/CNT1 *SCL28A2*/CNT2; *SCL28A3*/CNT3; *SLC29A1*/ENT1; *SLC29A2*/ENT2), the transporter with the highest level of expression was *SLC29A1* [31]. P-gp (*ABCB1*), encoded by the multidrug resistance-1 (MDR1) gene, is a drug efflux pump which belongs to the ATP-binding cassette (ABC) superfamily (ABCB subfamily). P-gp is expressed in different tissues including human breast tissue, although the downregulation of P-gp during lactation has been shown [31,32,33]. Our data show that MDR1 is expressed in hMECs, albeit at a low level. The functionality assay shows P_app_ values of fexofenadine similar to atenolol, suggesting limited P-gp transporter activity in the hMEC in vitro model. Multidrug resistance-related proteins (MRPs) constitute another subfamily of transporters (ABCC). Among the ABCC subfamily, the transcripts of MRP1-5 were detectable with a high level of expression. These data disagree with data related to human non-lactating cells, as MRP3 and MRP4 were not detectable [31]. CDFDA was used as a probe substrate for MRP-mediated transport and suggests low functionality of MRP in the hMEC in vitro model. Breast cancer resistance protein (BCRP/*ABCG2*) has been described to be one of the main transporters at the blood–milk barrier. Indeed, we showed a high level of protein expression in the primary culture of hMECs according to the high expression observed in the human mammary gland [34]. In contrast, we found a low functionality of BCRP using sulfasalazine as the probe substrate. Further research using alternative probe substrates and a concentration range might be needed to clarify the role of BCRP in the hMEC in vitro model. The absence of not only BCRP, but also OCT1/3 activity, might indicate that the current in vitro model represents a resting rather than lactating state [34]. Future efforts could be made to further differentiate the primary hMECs into a lactating state.

Overall, there was limited to no polarity in the apparent permeability of the probe substrates, i.e., transport rates were comparable in both directions. This suggests that passive diffusion is responsible for the transport of medicines across the hMEC in vitro model, and that saturable and/or active transport via membrane transporters was minimal [35,36]. A major limitation for the thorough characterization of an in vitro model for the blood–milk barrier is the rather limited knowledge on the in vivo expression of membrane transporters in humans. Quantitative liquid-chromatography coupled with a tandem mass spectrometry-based proteomics analysis of both the in vitro hMEC model and human breast tissue could be used to further clarify the role of the different transport routes in the blood–milk barrier.

It remains to be explored to what extent the permeability values determined in the in vitro hMEC model developed here can be used to predict the milk-to-plasma (M/P) ratio of (candidate) medicines. Importantly, other factors, such as the unbound fraction as well as the degree of ionization in plasma and milk have been demonstrated to influence this M/P ratio [35,36]. Overall, the results obtained here define a well-characterized human biorelevant in vitro model which could offer potential for rapid screening and an early assessment of drugs in a reproducible manner. The in vitro epithelial blood–milk barrier model could allow for the prediction of human milk drug distribution, providing quantitative data that inform researchers for better in vivo study designs, reducing animal use. In addition, in vitro permeability data show the potential to integrate the physiologically based pharmacokinetic PBPK model, enabling the prediction of the pharmacokinetic (PK) profile in human milk.

## 4. Materials and Methods

### 4.1. Chemicals and Reagents

MCF 10A (ATCC^®^ CRL10317™) and MCF-7 cells (ATCC) cells were a kind gift from prof. Fendt from the laboratory of cellular metabolism and metabolic regulation (VIB-KU Leuven). Dulbecco’s modified eagle medium/nutrient mixture F12 (DMEM/F-12, 31330038), horse serum (16050122), Dulbecco phosphate buffered saline (DPBS) with calcium and magnesium, fetal bovine serum (FBS), trypsin-EDTA, and antibiotic–antimycotic (anti-anti, 100×) (15240062) were purchased from Thermo Fisher Scientific (Waltham, MA, USA). Epithelial growth factor (AF-100-15) was purchased from Peprotech (London, UK). Hydrocortisone (H0888), cholera toxin, insulin (I9278), PMC42-LA cells (SCC139, Q2920330), Roswell Park memorial institute culture medium (RPMI-1640, R8758), β-estradiol (E2758), Dimethyl sulfoxide (DMSO), and FluoroshieldTM with 4′,6-diamidino-2-phenylindole (DAPI) histology mounting medium, were purchased from Sigma-Aldrich (St. Louis, MO, USA). FBS (BO 04-007-1A), penicillin/streptomycin (DE17-602E/12), Dulbecco’s modified eagle medium (DMEM, BE12-707F), and Hanks’ balanced salt solution were purchased from Westburg Life Sciences (Leusden, The Netherlands). Human mammary epithelial cells (hMECs, A10565 Lot # 2098293) were purchased from Life Technologies. Mammary epithelial cell growth medium (MECGM) consisted of mammary epithelial cell basal medium and a supplement mix containing 0.004 mL/mL bovine pituitary extract (BPE), 10 ng/mL human epithelial growth factor (hEGF), 5 μg/mL insulin, and 0.5 μg/mL hydrocortisone, purchased from Promo Cell (Heidelberg, Germany), and 1% anti-anti. 4-(2-hydroxyethyl)-1-piperazineethanesulfonic acid (HEPES) and atenolol (ACRO449550010) were purchased from VWR International (Radnor, PA, USA). Glucose was purchased from Thermo Fisher Scientific. TRI Reagent was purchased from Molecular Research Center Inc., Cincinnati, OH, USA and NucleoSpin RNA II kit from Macherey-Nagel GmbH & Co. KG, Düren, Germany. RT2 First Stand Kit, RT2 SYBR Green qPCR Mastermix, RT2 Profiler™ PCR array pig drug transporters (Cat. No. PAHS-070ZD), and RT2 Profiler™ PCR array human drug transporters (Cat. No. PASS-070ZD) were purchased from Qiagen, Hilden, Germany. FBS (F9665-500ML) was purchased from Merck Chemicals Ltd. (Darmstadt, Germany) Fexofenadine hydrochloride (F9427), 5(6)-Carboxy-2,7-dichlorofluorescein diacetate (CDFDA, 21884), sulfasalazine (SIGMA:S0883), methotrexate hydrate (06563), and propranolol hydrochloride (P0884) were purchased from Merck Life Science BV (Hoeilaart, Belgium). DMSO (D/4120/PB08) was purchased from Acros Organics NV (Geel, Belgium). Metformin hydrochloride (257998) was purchased from J&K Scientific BV (Lommel, Belgium). The antibodies used for the immunofluorescence (IF) and flow cytometry analysis (FC) are listed in Table 2.

### 4.2. Culture of MCF-10A Cells

MCF-10A cells were expanded according to the protocol from the BRUGGE lab, department of cell biology, Harvard medical school (https://brugge.med.harvard.edu/protocols. Accessed 9 March 2021). Briefly, cells were cultured in DMEM/F-12 with addition of 5% heat inactivated horse serum, 20 ng/mL epithelial growth factor, 0.5 µg/mL hydrocortisone, 100 ng/mL cholera toxin, 10 µg/mL insulin and 1% penicillin/streptomycin. MCF-10A cells were expanded at a density of 1.5 × 10^4^ cells/cm^2^ in normal cell culture flasks (CELLSTAR^®^). Medium was changed every 2–3 days.

### 4.3. Culture of PMC42-LA Cells

PMC42-LA cells were expanded according to the protocol provided by Sigma-Aldrich. Briefly, PMC42-LA cells were cultured in RPMI-1640, with addition of 10% FBS and 1% penicillin/streptomycin. PMC42-LA cells were expanded at a density of 1 × 10^6^ cells/cm^2^ in normal cell culture flasks (CELLSTAR^®^). Medium was changed every 2–3 days.

### 4.4. Culture of MCF-7 Cells

MCF-7 cells were expanded based on the protocol from the laboratory of cellular metabolism and metabolic regulation [37]. Briefly, MCF-7 cells were cultured in DMEM, with addition of 20 µg/mL β-estradiol, 10% FBS, and 1% penicillin/streptomycin. β-estradiol was omitted from the culture medium at a later stage, as it was suspected to interfere with the formation of a tight monolayer. MCF-7 cells were expanded at a density of 4 × 10^4^ cells/cm^2^ in normal cell culture flasks (CELLSTAR^®^). Medium was changed every 2–3 days.

### 4.5. Culture of hMECs

Primary cultures of hMECs were purchased by Life Technologies (A10565 Lot. 2098293; Gibco, MD, USA). Ethical approval for acquiring, passaging, storing, and culturing hMECs was obtained from the Ethics Committee Research (EC Research) UZ/KU, Leuven, Belgium (reference number S67546). hMECs were cultured, as per data sheet indication, in MECGM, which consisted of mammary epithelial cell basal medium, with addition of 0.0004 mL/mL BPE, 10 ng/mL hEGF, 5 µg/mL insulin, 0.5 µg/mL hydrocortisone, and 1% anti-anti. Cells were seeded at a density of 5 × 10^3^ cells/cm^2^ in T75 primary culture flasks (Corning PRIMARIA^TM^) and cultured until 80% confluence, then the cells were detached with trypsin-EDTA 1× solution, counted, and expanded until passage 10. Doubling time was calculated as previously described [38], aliquots of 5 × 10^5^ cells were cryopreserved in 1 mL of freezing medium (90% FBS and 10% DMSO). Cell morphology was monitored using an inverted optical microscope equipped with an inverted Eclipse Microscope (TS100) and a digital C-Mount Nikon photocamera (TP3100) was used to define the cell morphology.

### 4.6. Barrier Function

The formation of a tight cellular barrier is essential to evaluate the in vitro permeability of medicines. The barrier function of the mammary epithelial cell lines and primary mammary epithelial cells cultured on permeable supports was evaluated by measuring the transepithelial electrical resistance (TEER) and transport of sodium fluorescein, as previously described [16,17]. The explored culture conditions (Supplementary Table S1) include the following: (i) a range of passage number and seeding densities; (ii) different permeable support material, pore size, surface area, and coatings; and (iii) a variety of differentiation media (e.g., full growth culture medium, addition of serum or prolactin, removal of epidermal growth factor, beta-estradiol, or cholera toxin). MCF-10A cells were seeded at a density of 1.5 × 10^3^ to 2 × 10^5^cells/cm^2^ on polycarbonate (PC) or polyethylene terephthalate (PET) transwell inserts with a pore size of 0.4 or 3.0 µm and a surface area of 0.3 cm^2^ or 1.12 cm^2^. The differentiation medium used for transwell culture was full growth medium with horse serum concentrations of 1%, 5%, or 10%. In addition, serum-free medium or medium without cholera toxin was explored. The medium was changed daily or every 2–3 days. PMC42-LA cells were seeded at a density of 5 × 10^4^ to 2 × 10^5^ cells/cm^2^ on PET transwell inserts with a pore size of 0.4 or 3.0 µm and a surface area of 0.3 cm^2^. The inserts were uncoated or coated with Matrigel (0.2–1 mg/mL). The differentiation medium used for transwell culture was full growth medium with fetal bovine serum concentrations of 5% or 10%. MCF-7 cells were seeded at a density of 4 × 10^4^ to 6 × 10^5^ cells/cm^2^ on PET transwell inserts with a pore size of 0.4 or 3.0 µm and a surface area of 0.3 cm^2^. The differentiation medium used for transwell culture was either full growth medium or medium without beta-estradiol, both with fetal bovine serum concentrations of 5% or 10%. Primary cultures of hMECs (P8) were seeded at a density of 3.3 × 10^5^ cells/cm^2^ on PET transwell inserts with a pore size of 0.4 µm and a surface area of 0.3 cm^2^.

### 4.7. Transepithelial Electrical Resistance (TEER)

Resistance in function of culture time was measured with an Epithelial Volt/Ohm Meter 2 (EVOM2, World Precision Instruments) at 37 °C. TEER (Ω × cm^2^) was calculated based on the difference between the total resistance and the mean resistance for a permeable support without cells, multiplied with the surface area of the permeable support (M_area_, cm^2^) (Equation (1)). Data collected over time were analyzed by using Excel (Microsoft 365 MSO, version 2409 build 16.0.18025.20160) and R studio software (version 4.2.2).

(1) $$Transepithelial\;electrical\;resistance\;\left(\Omega *{cm}^{2}\right)\;\;={(Resistance}_{total}(\Omega )-{Resistance}_{blank}(\Omega ))*{Area}_{membrane}({cm}^{2})$$

### 4.8. Sodium Fluorescein Transport

The leakage of sodium fluorescein (2.66 mM) through the monolayer of cells grown on the permeable supports was measured after one hour of incubation at 37 °C under gentle shaking. The buffer (pH 7.4) consisted of Hanks’ balanced salt solution, with addition of 10 mM HEPES and 25 mM glucose. The sodium fluorescein leakage was measured via fluorescence intensity (λ = 490/524 nm) using a Tecan Infinite M200 plate reader (Tecan Group Ltd., Männedorf, Austria). The buffer was used as a blank, and a calibration curve was prepared with concentrations of sodium fluorescein ranging from 207.8 nM (0.0078%) to 3.325 µM (0.125%). The percentage of sodium fluorescein transport (Equation (2)) was the concentration of sodium fluorescein in the sample after one hour relative to the donor concentration of sodium fluorescein, corrected for the respective volumes in the basolateral and apical compartment. Data collected were analyzed by using Excel and R studio software (version 4.2.2).

(2) $${Transport\;}_{sodium\;fluorescein}\left(\%\right)=\frac{{Concentration}_{sample}(mol/L)}{{Concentration}_{donor}(mol/L)}\times \frac{{Volume}_{basolateral}\;(L)}{{Volume}_{apical}(L)}\times 100\%$$

### 4.9. Immunophenotypic Characterization by Flow Cytometry (FC) Analysis

The expression of epithelial cadherin (E-CAD) and cytokeratin 18 (CK18) in single hMECs was evaluated using FC, as described previously [16]. In brief, cells were fixed in paraformaldehyde and permeabilized in methanol. They were then incubated with either anti-E-CAD or anti-CK18 antibodies (listed in Table 2). Negative controls were obtained by omitting primary antibodies. Cell analysis was performed using the MacsQuant Analyzer10 (Miltenyi Biotec, Bergisch Gladbach, Germany) and the Flowlogic™ software (version 7.1, Inivai Technologies, Mentone, VIC, Australia). Fluorescent staining intensity was determined by comparing the median intensity fluorescence (MFI) of the negative control with that of single stained cells. Data were analyzed with the Student’s *t* test comparing the MFI of the negative control and the MFI of single stained cells (*p* < 0.05).

### 4.10. Immunohistochemical Staining

The hMECs, cultured on a transparent permeable support of membrane pore size 0.4 µm and area of 0.3 cm^2^, after 35 days of culture, were assessed by checking the presence of epithelial markers (E-CAD and CK18) and tight junctions (ZO-1 and OCL). The cells were fixed and permeabilized following the procedure described previously [17,39]. The cells were incubated overnight at 4 °C with the primary antibodies (Table 2) diluted in DPBS. After being rinsed in DPBS (3 times 10 min each), the cells were incubated with fluorochrome-labeled secondary antisera diluted in DPBS 1 h RT (Table 2). After 3 washes (10 min each) in DPBS, the membrane was detached from the transwell and placed on slide. Mounting medium with DAPI to counterstain the nuclei was added on slide and on top of the membrane. Images acquisition was obtained using a Zeiss Axioplan 2 microscope, equipped with a Hamamatsu ORCA-spark CMOS C11440-36U camera (Göttingen, Germany). The setup was controlled by μManager (version 1.4.23).

### 4.11. mRNA Expression of Membrane Transporters

RNA extraction was performed using TRI Reagent and the NucleoSpin RNA II kit, as previously described [17]. After spectrophotometric quantification (DeNovix DS-11, DeNovix Inc., Wilmington, NC, USA) total RNA (500 ng) was reverse-transcribed to cDNA using the RT2 First Stand Kit. Then, RT2 Profiler™ PCR Array Human Drug Transporters were performed as previously described [16,40]. The gene expression of 84 human transporter genes was analyzed using the ΔCt method (mean Ct Reference Gene (R.G.)—Ct Interest Gene (I.G.)), according to the RT2 Profiler PCR Array Handbook. The reference genes used were as follows: Beta Actin (ACTB); Beta-2-microglobulin (B2M), Glyceraldehyde-3-phosphate dehydrogenase (GAPDH), Hypoxanthine phosphoribosyltransferase (HPR1), and Ribosomal protein, large P0 (RPLP0).

### 4.12. Functional Assay of Membrane Transporters

The function of membrane transporters in primary hMECs was evaluated using probe substrates. The hMECs were cultured on 24-well polyethylene terephthalate transwell inserts (Falcon, 353095) with a surface area of 0.3 cm^2^ and pore sizes of 0.4 µm at a density of 3 × 10^5^ cells/cm^2^. The differentiation medium used MECM, where BPE was replaced by 10% of FBS. The medium was changed every 2–3 days. The bidirectional permeability experiments were performed after 35 days, when a tight barrier was obtained on the transwell insert. One day before the experiments, the medium was changed (0.3 mL apical and 0.72 mL basolateral). TEER was measured before the start of the experiments.

The functionality of hMECs was analyzed by selecting known probe substrates for the transporters that are considered involved in drug medicine transport across the mammary epithelium. Stock solutions (100 mM) of fexofenadine hydrochloride for P-gp (*ABCB1*) function, CDFDA for MRP2/3 (*ABCC2/3*) function, sulfasalazine for BCRP (*ABCG2*) function, and methotrexate hydrate for OAT (*SLC22A6/7/8/9*) function were prepared in DMSO. For paracellular transport, a stock solution (50 mM) of atenolol was prepared in 50:50 ultrapure water–DMSO. For passive transcellular transport, a stock solution (50 mM) of propranolol hydrochloride was prepared in DMSO. For OCT (*SLC22A1/2/3*), a stock solution (100 mM) of metformin hydrochloride (257998) was prepared in ultrapure water. Stock solutions were stored at −20 °C for a maximum of 6 months.

Donor solutions with a mixture of probe substrates were prepared in differentiation medium as follows:

A. atenolol, propranolol and fexofenadine;
B. CDFDA and sulfasalazine;
C. metformin and methotrexate.

Differentiation medium was added to the receptor compartment, and the donor solution was added to the donor compartment to obtain a final concentration of 50 µM of each probe substrate in differentiation medium (0.2% DMSO). Final volumes were 0.5 mL in the apical compartment and 1.2 mL in the basolateral compartment. A sample was taken from each dosing solution. Bidirectional transport experiments were performed for 2 h at 37 °C under gentle shaking (120 rpm). Three independent experiments were performed, with each three or six inserts for each donor solution mixture. Receptor compartment samples (half of the compartment volume) were taken at 0.5, 1, and 1.2 h, and differentiation medium was added to replace the sample volume. The dilutions were taken into account when calculating the cumulative amount transported. At the end of the experiment (2 h), samples were taken from the receptor and donor compartments. The integrity of the epithelial cell layer(s) was confirmed after the transport experiments via measurement of transepithelial electrical resistance (TEER) and sodium fluorescein leakage, as described previously for the determination of the barrier function.

### 4.13. Data Analysis on Transporters Functionality

The apparent permeability coefficient (P_app_) was calculated using Equation (3). Samples below the limit of quantification were not used when calculating the slope of the cumulative amount transport curve. The linearity of the cumulative amount transported curve was assessed, and the initial points were removed until the linearity threshold was reached (R^2^ ≥ 0.970). Apparent permeability was only calculated if more than 2 time points remained. The P_app_ values were calculated as follows:

(3) $${Permeability}_{apparent}\left(\frac{cm}{s}\right)=\frac{dQ}{dt}(\frac{signal}{s})*\frac{1}{{Area}_{membrane}({cm}^{2})*{Concentration}_{donor}(\frac{signal}{mL})}$$

The signal of the area-under-the-curve of the compound peak was obtained after the liquid chromatography with tandem mass spectrometry (LC-MSMS) bioanalysis and dilutions made during sample preparation and the injection volume were corrected. Data collected were analyzed by using Excel for calculations of apparent permeability coefficients. R studio software (version 4.2.2) was used to generate the graphs.

### 4.14. Bioanalysis

Three analytical methods were used for different groups of probe substrates: (1) atenolol, propranolol, and fexofenadine, (2) sulfasalazine and CDF, and (3) metformin and methotrexate. CDFDA is rapidly hydrolyzed to 5(6)-carboxy-2,7-dichlorofluorescein (CDF) by intracellular esterases. Hence, CDF was determined to indicate MRP-mediated transport. All reagents used were of UHPLC or MS grade.

### 4.15. Sample Preparation

Extraction was achieved by the protein precipitation method with different precipitating agents. Samples containing atenolol, propranolol, fexofenadine, CDF, and sulfasalazine were precipitated at a 1:4 ratio of sample to methanol. Precipitated sample solutions were centrifuged at 12,000 rpm for 10 min under 21 °C. The 100 µL of supernatant was mixed with 100 µL of water to be used as an injection solution for analysis. Other samples with metformin and methotrexate were extracted with a similar procedure but used 0.1% formic acid-containing acetonitrile as precipitating agents. The injection solutions consisted of 100 µL of supernatant and 100 µL of acetonitrile–water–formic acid (60:40:0.1, *v*/*v*/*v*).

### 4.16. Liquid Chromatographic and Mass Spectrometric Parameters

Analyses were conducted with a Shimadzu UHPLC-MS system (Kyoto, Kyoto, Japan) consisting of two pumps (LC-30AD), an autosampler (ISL-30AC), a column over CTO-20AC, and an LCMS-8050 triple quadrupole mass spectrometer equipped with electrospray ionization (ESI). LabSolutions software version 6.86 was used for system control and data processes. LC separation of analyte group (1), (2), and (3) were performed on Phenomenex (Torrance, CA, USA) columns: Luna^®^ Omega Polar C18 (50 × 2.1 mm, 1.6 µm), Kinetex^®^ F5 (100 × 2.1 mm, 1.7 µm), and Kinetex^®^ HILIC (100 × 2.1 mm, 2.6 µm), respectively. Columns were kept at 40 °C during analysis. The mobile phase consisted of A) 10 mM ammonium formate in water with 0.1% formic acid and B) 0.1% formic acid in acetonitrile; gradient programs of methods are detailed in Table 3. Samples were kept at 15 °C in the autosampler during the whole analysis. The ionization of analytes was operated at positive mode, except for CDF at negative mode. The ESI voltage was 4 kV for all analytes. Other interface parameters were set as follows: interface temperature at 300 °C, nebulizing gas (nitrogen) flow at 3 L/min, heating gas (dry air) flow and drying gas (nitrogen) flow at 10 L/min, desolvation line temperature at 250 °C, and heat block temperature 300 °C. Argon gas was applied to the collision-induced dissociation of precursor ions. The two most abundant product ions of each analyte were selected to be the quantifier and qualifier. The multiple reaction monitoring (MRM) transitions of analytes and corresponding collision energies are shown in Table 4.

## 5. Conclusions

In conclusion, primary hMECs were able to reproducibly form an epithelial barrier in vitro and the ability to distinguish between passive paracellular and transcellular transport routes, making them a promising in vitro model for the blood–milk barrier. This in vitro tool offers the potential to generate evidence supporting the use of medicines during lactation at an early stage of drug development. Further characterization and application remain necessary to explore the predictive potential of the proposed in vitro model. Ongoing efforts within the ConcePTION project aim to apply the developed in vitro model for 10 model medicines, and to compare the obtained permeabilities with permeability generated using a minipig in vitro model or established in vitro models (e.g., Caco-2).

## Figures and Tables

**Table 1:** Median apparent permeabilities of probe substrates for transport routes for three independent experiments (*n* = 6, *n* = 6, *n* = 3).

Probe Substrate	Permeability Direction	Median Apparent Permeability (10^−6^ cm/s)
Atenolol	Apical-to-basolateral	0.38
		0.03
		0.28
	Basolateral-to-apical	0.63
		0.41
		0.51
CDF(DA)	Apical-to-basolateral	-- ^(a)^
		±0.17 ^(b)^
		-- ^(a)^
	Basolateral-to-apical	-- ^(a)^
		0.17
		0.31
Fexofenadine	Apical-to-basolateral	0.28
		0.11
		0.23
	Basolateral-to-apical	0.44
		0.32
		0.41
Metformin	Apical-to-basolateral	0.86
		≤0.11 ^(c)^
		≤0.11 ^(c)^
	Basolateral-to-apical	0.85
		≤0.05 ^(c)^
		≤0.05 ^(c)^
Methotrexate	Apical-to-basolateral	0.20
		≤0.11 ^(c)^
		≤0.11 ^(c)^
	Basolateral-to-apical	0.35
		≤0.05 ^(c)^
		≤0.05 ^(c)^
Propranolol	Apical-to-basolateral	7.65
		15.73
		18.18
	Basolateral-to-apical	5.73
		11.62
		14.30
Sulfasalazine	Apical-to-basolateral	-- ^(a)^
		≤0.11 ^(c)^
		-- ^(a)^
	Basolateral-to-apical	0.43
		0.19
		0.23

^(a)^ Permeability coefficient could not be determined; ^(b)^ permeability coefficient was estimated from two time points, as other time points were below the quantification limit; ^(c)^ permeability coefficient could not be determined as samples were below the quantification limit. Abbreviations: CDF(DA), 5(6)-Carboxy-2,7-dichlorofluorescein diacetate; CDF, 5(6)-Carboxy-2,7-dichlorofluorescein.

**Table 2:** Antibodies used for the immunofluorescence (IF) and flow cytometry (FC) analysis.

*Antibody*	*P. Number*	*Specie*	*Supplier*	*Application*
*Primary*				
Anti CK	GA053	Mouse	Agilent Dako	IF: 1:150
Alexa Fluor 647 anti E-cadherin	147307	Rat	BioLegend	IF: 1:50
Alexa Fluor 488 anti Ck-18	Ab187573	Mouse	Abcam	IF: 5 μg/mL
Anti ZO-1	61-7300	Rabbit	Thermo Fisher	IF: 1:100
Anti OCL	sc-133255	Mouse	Santa Cruz Biotechnology	IF: 1:50
Brilliant Violet 421^TM^ anti E-cadherin	147319	Rat	BioLegend	FC: 17 μL/10^6^
FITC anti-cytokeratin 18	ab52459	Mouse	Abcam	FC: 10 μL/10^6^
*Secondary*				
Anti-mouse IgG-Alexa Fluor 594	A11032	Goat	Thermo Fisher	IF: 5 µg/mL
Anti-rabbit IgG-Alexa Fluor 488	A11034	Goat	Thermo Fisher	IF: 5 µg/mL

Abbreviations: CK, cytokeratin; FITC, Fluorescein isothiocyanate; IgG, immunoglobulin G; OCL, Occludin; ZO-1, Zonula Occludens-1.

**Table 3:** Gradient program of mobile phase used in three methods.

Analyte Group	Atenolol, Propranolol and Fexofenadine	Sulfasalazine and CDF	Metformin and Methotrexate
Gradient program	0–0.5 min, 5% B 0.5–3 min, 5–50% B 3–4 min, 50% B 4–4.2 min, 50–5% B 4.2–6 min, 5% B	0–0.5 min, 30% B 0.5–2 min, 30–60% B 2–3 min, 60% B 3–3.5 min, 60–30% B 3.5–6 min, 30% B	Isocratic running at 80% B.

Abbreviations: CDF, 5(6)-Carboxy-2′,7′-dichlorofluorescein.

**Table 4:** Multiple reaction monitoring (MRM) parameters of analytes.

Analyte	MRM Transition: (Quantifier and Qualifier)	Collision Energy
Atenolol	267.05 → 145.15	−26
	267.05 → 190.2	−19
Propranolol	260.15 → 116.2	−17
	260.15 → 155.2	−25
Fexofenadine	502.3 → 466.4	−28
	502.3 → 171.0	−37
Sulfasalazine	399.0 → 317.05	−22
	399.0 → 381.15	−18
CDF	443.0 → 319.1	30
	443.0 → 363.1	23
Metformin	130.2 → 60.2	−14
	130.2 → 70.9	−21
Methotrexate	455.2 → 175.15	−31
	455.2 → 308.25	−23

Abbreviations: CDF, 5(6)-Carboxy-2,7-dichlorofluorescein; MRM, Multiple reaction monitoring.

## Data Availability

Data related to doubling time, flow cytometry analysis, and RT2 array Profiler are available on AMSActa Institutional Research Repository by AlmaDL University of Bologna Digital Library: https://amsacta.unibo.it/id/eprint/7808 (accessed on 22 October 2024). Data related to barrier integrity and functionality of transport rates are available on KU Leuven Research Data Repository: https://www.kuleuven.be/rdm/en/rdr (accessed on 22 October 2024).

## Supplementary Materials

The following supporting information can be downloaded at: https://www.mdpi.com/article/10.3390/ijms252111454/s1.
