# Supplementary material for: Development and Characterization of a Human Mammary Epithelial Cell Culture Model for the Blood–Milk Barrier—A Contribution from the ConcePTION Project
Source: Int J Mol Sci. 2024 Oct 25;25(21):11454. doi: 10.3390/ijms252111454 (PMC11546117; doi:10.3390/ijms252111454)
Supplement: Supplementary file 1 [file ijms-25-11454-s001.zip › Supplementary material.pdf]

Representative pictures of immortalized cell lines (MCF-7, MCF-10A, PMC42-LA)

Figure S1. Representative picture (10X magnification) of a) MCF-7 cell line passage 9 at day 5 of culture in cell culture flasks; b) MCF-7 cell line passage 12 at day 4 of transwell culture; c) MCF-10A cell line passage 11 at day 3 of culture in cell culture flasks; d) MCF-10A cell line passage 15 at day 8 of transwell culture; e) PMC42-LA cell line passage 4 at day 3 of culture in cell culture flasks; f) PMC42-LA cell line passage 4 at day 1 of transwell culture

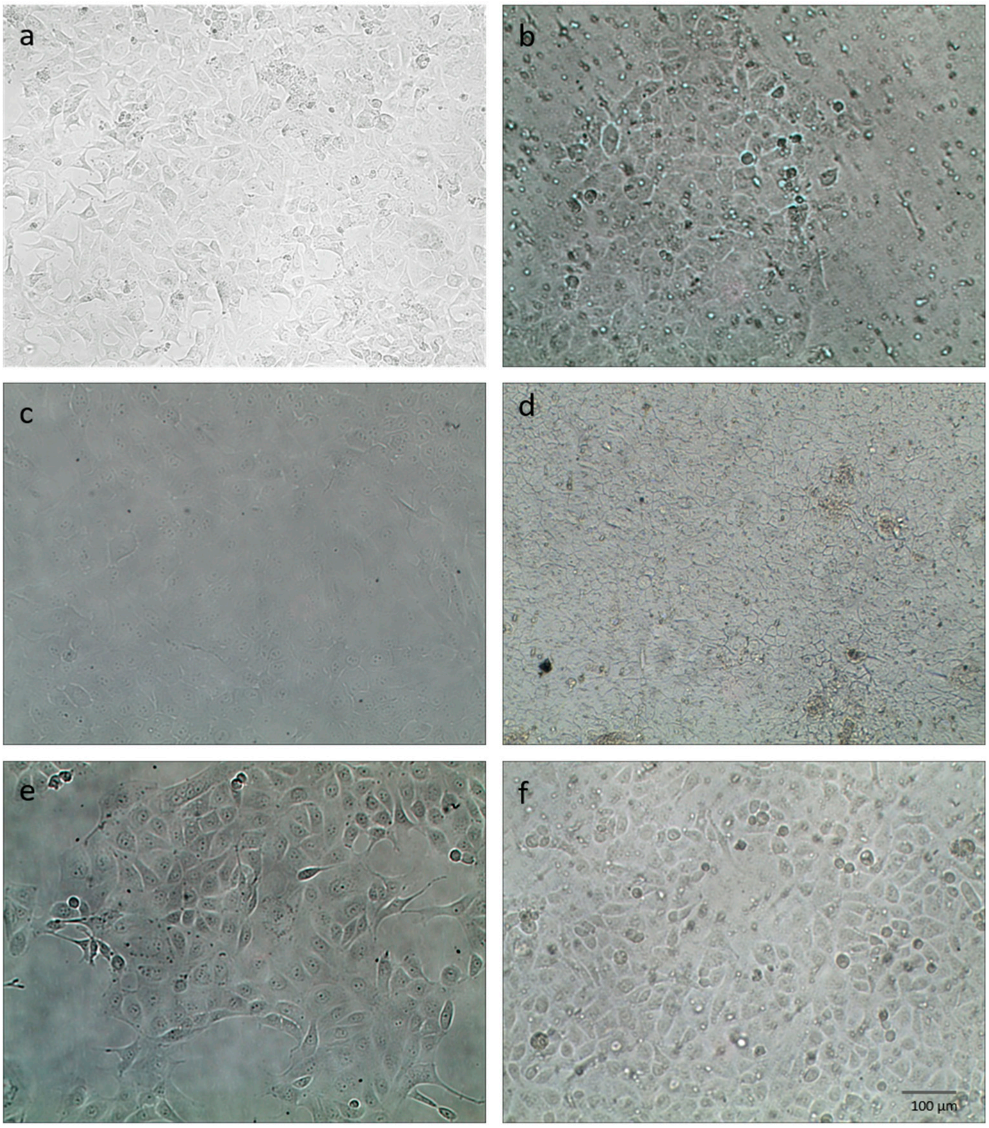

Overview of TEER and sodium fluorescein measurements for primary hMECs and human cell lines

Table S1. Overview of transepithelial electrical resistance (TEER, $\Omega \times cm^2$) and sodium fluorescein measurements for primary hMECs (hMECs) and human cell lines (MCF-7, MCF-10A and PMC42-LA)
